# Home energy efficiency and radon: An observational study

**DOI:** 10.1111/ina.12575

**Published:** 2019-06-18

**Authors:** Phil Symonds, David Rees, Zornitza Daraktchieva, Neil McColl, Jane Bradley, Ian Hamilton, Michael Davies

**Affiliations:** ^1^ UCL Institute for Environmental Design and Engineering Central House London UK; ^2^ Dosimetry Services Department, Public Health England Centre for Radiation Chemical and Environmental Hazards Chilton Oxon UK; ^3^ UCL Energy Institute Central House London UK

**Keywords:** big UK dataset, home energy efficiency, indoor air quality, longitudinal study, radon, ventilation

## Abstract

Exposure to radon gas is the second leading cause of lung cancer worldwide behind smoking. Changing the energy characteristics of a dwelling can influence both its thermal and ventilative properties, which can affect indoor air quality. This study uses radon measurements made in 470 689 UK homes between 1980 and 2015, linked to dwelling information contained within the Home Energy Efficiency Database (HEED). The linked dataset, the largest of its kind, was used to analyze the association of housing and energy performance characteristics with indoor radon concentrations in the UK. The findings show that energy efficiency measures that increase the airtightness of properties are observed to have an adverse association with indoor radon levels. Homes with double glazing installed had radon measurements with a significantly higher geometric mean, 67% (95% CI: 44, 89) greater than those without a recorded fabric retrofit. Those with loft insulation (47%, 95% CI: 26, 69) and wall insulation (32%, 95% CI: 11, 53) were also found to have higher radon readings. Improving the energy performance of the UK's housing stock is vital in meeting carbon emission reduction targets. However, compromising indoor air quality must be avoided through careful assessment and implementation practices.

**Practical Implications**

- There is a need to ensure that appropriate measures are put in place to assess and address possible increases in radon exposure post‐intervention.
- While energy efficiency measures are likely to provide a net benefit in terms of energy savings and warmer homes, care should to be taken to mitigate against reductions in air quality when installing interventions that increase the airtightness of homes.
- Increases in integrated population exposure to radon will lead to a rise in radon‐related lung cancer rates.
- Energy efficiency interventions in radon‐affected areas should be coupled with radon risk assessment strategies and monitoring to check that radon levels are not negatively impacted.
- Efforts should be made, where necessary, to reduce high indoor radon concentrations to below the Public Health England target level of 100 Bq/m^3^.

## 1 INTRODUCTION

Radon is a naturally occurring radioactive gas and has been identified as the second leading cause of lung cancer worldwide after tobacco smoking. It is estimated to cause between 3% and 14% of lung cancer deaths depending on average radon levels and smoking prevalence.^1^ In the UK, 1100 annual deaths have been attributed to radon exposure in homes in a Public Health England (PHE) report,^2^ while a recent international study put the UK figure at 2858 (95% CI: 219, 9419).^3^ Radon is emitted from all soil and rock types at various concentrations and presents a continuous source of human radiation exposure,^4^ though quick dilution in the atmosphere leads to low concentrations in open spaces. In enclosed spaces, however, concentrations can become relatively high as it enters through gaps and cracks in suspended floors, construction joints, or walls.^5^

In accordance with the Paris Climate Change Agreement, governments are committed to limiting global average temperature rise to well below 2°C above pre‐industrial levels.^6^ To achieve this target, a variety of measures are required to reduce greenhouse gas (GHG) emissions. These measures can be made both at source through, for example, increased generation of renewable energy and at point of use (eg, through home energy efficiency (HEE) measures). The UK government has pledged to reduce GHG emissions by 80% (from the 1990 baseline) by 2050.^7^ The domestic housing stock is one of the areas targeted, with HEE measures incentivized through schemes such as the Energy Company Obligation. A large body of evidence has been amassed in recent years on how such measures might impact on the health of building occupants. Hamilton et al^8^ have shown that such measures (if installed according to building regulations) can help reduce winter cold and indoor pollutant exposure resulting in a net gain in quality‐adjusted life years. However, HEE measures installed without adequate purpose provided ventilation may result in adverse health impacts due to increased exposure to internally produced pollutants.^9^

As with other internally produced pollutants, the air tightening of buildings may inhibit radon from leaving the indoor environment or increase the stack effect, causing it to accumulate.^10^ A recent modeling study indicated that increasing the airtightness of English homes (without providing compensatory ventilation) would increase indoor radon concentrations by around 60%, resulting in an annual burden of 4700 life years lost and 278 deaths (at peak) per year.^11^ While earlier empirical studies have investigated the impact of dwelling characteristics on indoor radon concentration measurements, they relied on smaller samples.^12^ The interaction between indoor radon levels and the presence of energy efficiency attributes of dwellings has been studied previously, with Gunby et al using data from a national radon survey.^13^, ^14^ Associations were identified between indoor radon levels and the presence of double glazing and downdraft proofing using data from around 2000 dwellings with property information provided by the occupiers. This paper uses a substantially larger dataset of greater coverage and over a longer timescale than previous studies. This allows the relationship between various dwelling characteristics such as HEE interventions and indoor radon concentrations to be empirically derived for a UK setting.

## 2 METHODS

This study involves the analysis of radon measurements in approximately 470 000 UK homes held by PHE, matched to dwelling characteristics recorded in the Homes Energy Efficiency Database (HEED). There were two main components to this analysis:

- Dataset matching and processing.
- Statistical analysis and interpretation.

The aim of this study is to investigate any relationships that may exist between dwelling characteristics and indoor radon concentrations with a particular focus on the impact of energy efficiency interventions which modify the building envelope. The addition of glazing, loft and wall insulation, and downdraft proofing are considered.

### 2.1 Datasets, matching and processing

#### 2.1.1 Indoor radon measurements

Public Health England holds over 525 000 radon measurements recorded in UK homes made over the period between 1980 and 2015. These radon measurements were collected over several measurement campaigns conducted by PHE (and previously the Health Protection Agency [HPA] and National Radiological Protection Board [NRPB]) for a variety of purposes. All valid radon measurements made by PHE are in the database. Since the database was established, the main sources have been, national and regional surveys, aimed at establishing the distribution of indoor radon levels in the UK; targeted programs, undertaken in areas of known higher radon risk and often including offers of free radon tests for householders; research programs investigating specific aspects of indoor radon; and radon measurements purchased by individual householders, landlords, and social housing providers. In some of the above cases, there is a deliberate bias toward obtaining measurements from areas of higher radon risk since that is where most of the higher individual exposures and risks are incurred and where intervention to reduce radon is most likely to be required. The dataset therefore has a known, deliberate bias toward high radon areas but includes over 150 000 radon measurements made in areas of lowest radon risk (outside “radon‐affected areas”).

**Figure 1:**
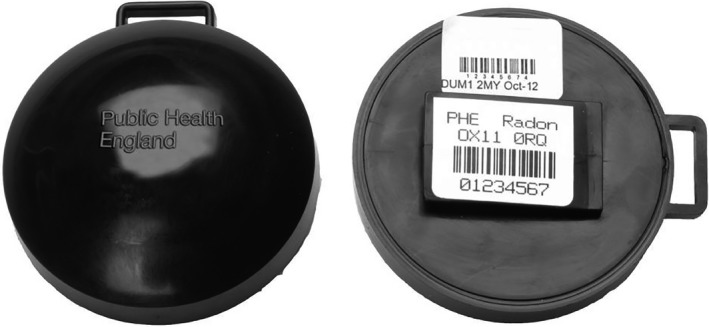
Passive monitors used by Public Health England to measure indoor radon concentrations. © Crown Copyright, 2013. Public Health England

The measurement procedure is reported in greater detail elsewhere.^15^ Briefly, measurements are usually made by two passive radon detectors (shown in Figure 1), placed by a member of the household (in accordance with instructions) in both the living room and an occupied bedroom. The detectors are left in place for three months and then returned to PHE who calculate an annual average household radon exposure (Bq/m^3^), which reflects typical occupancy patterns and seasonal corrections.^16^ At the radon Action Level (200 Bq/m^3^), an average 3‐month measurement is expected to have an uncertainty no greater than 15%, while for measurements in the ranges 46‐140 and 460‐1400 Bq/m^3^, the acceptable uncertainty is 25%,^17^ which includes uncertainties relating to the occupancy patterns and seasonal corrections.

#### 2.1.2 The homes energy efficiency database

The version of HEED used in this study comprises information on approximately 16.4 million UK dwellings and includes house‐level characteristics such as age and type (eg, detached and semi‐detached). Uptake of HEE measures is also included such as the installation of loft and wall insulation, boiler replacement, downdraft proofing, and the addition of double glazing. The data are broadly representative of the English housing stock, although flats are underrepresented.^18^ There are also substantial amounts of data missing in HEED.^19^ Information within the database used was compiled by the Energy Savings Trust and contains data collected between 1993 and 2016 from multiple sources; installers, industry accreditation bodies, energy suppliers, government‐funded programs, local authorities, and home surveys.^20^ Homes which have had multiple HEE interventions have multiple entries within HEED, meaning that energy efficiency retrofits can be tracked over time.

#### 2.1.3 Data matching and processing

Radon measurements were corrected for average outdoor radon concentrations by subtracting 4 Bq/m^3^ from all indoor measurements. Negative measurements were removed from any subsequent analysis. The radon and HEED datasets were matched using the postal address of the property. After the matching process, the address of the property was removed and anonymized to leave postcode district (the first four characters). The match resulted in a sample size of 470 689 homes. For homes with multiple radon measurements (some 20 000 homes), the match was made to the earliest measurement chronologically. This allowed the analysis to focus on HEE, since a second radon measurement typically follows radon mitigation measures applied to the home. The dwelling postcode district was also used to match to urban/rural classification (rucomb) using Office for National Statistics data.^21^

**Figure 2:**
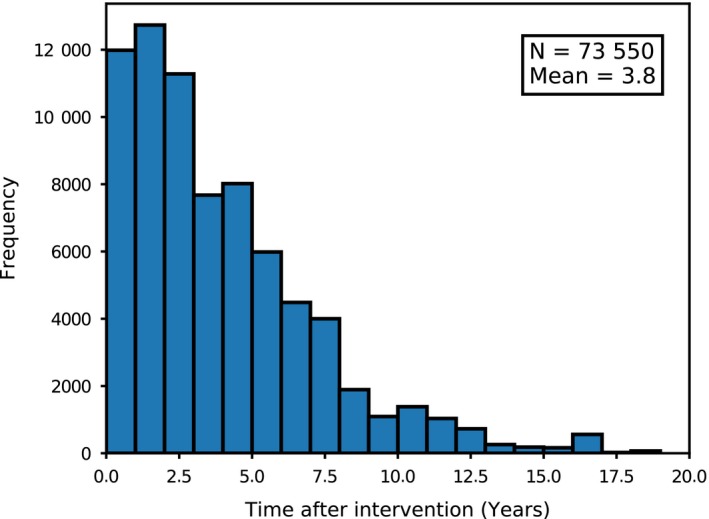
Time in years that a radon measurement follows the most recent energy efficiency intervention made in a home

Processing of the matched radon‐HEED data was performed, such that only HEE interventions made prior to a radon measurement were classified as retrofit data. Given that the radon measurement program began 13 years before HEED was initiated, only 15.6% (73 550) of the radon measurements follow any HEE intervention. Of these, the radon measurement follows the most recent HEED entry by an average of 3.8 years (1392.5 days). Figure 2 presents a histogram of the time in years after which the radon measurement followed a HEE retrofit. For cases where a radon measurement precedes the most recent HEE intervention, time‐invariant information (such as dwelling age and type) is used. However, information concerning HEE (such as wall insulation type) is treated as “missing data.”

### 2.2 Statistical analysis

Radon concentrations in homes are generally observed to be log‐normally distributed.^22^ Geometric means and standard deviations of indoor radon concentration have been calculated within groups of data to estimate radon variability between various dwelling characteristics. The following dwelling characteristics have been studied:

1. Those considered invariant with time:
  - Dwelling type.
  - Dwelling age.
  - Wall type.
  - Number of bedrooms.
  - Tenure.
  - Government Office Region (GOR).
  - Urban/rural class.
2. HEE measures:
  - Wall insulation type.
  - Presence of downdraft stripping/proofing.
  - Loft insulation level.
  - Glazing type.
  - Heating system type.

This paper focuses on HEE measures that modify the building envelope (fabric interventions) with results regarding heating system shown in Appendix S1. The data allowed for analysis of the specific HEE measures (eg, the thickness of loft insulation) and also the binary condition of whether a HEE measure had been installed or not, and their combination, and association with average radon levels.

## 3 RESULTS

**Figure 3:**
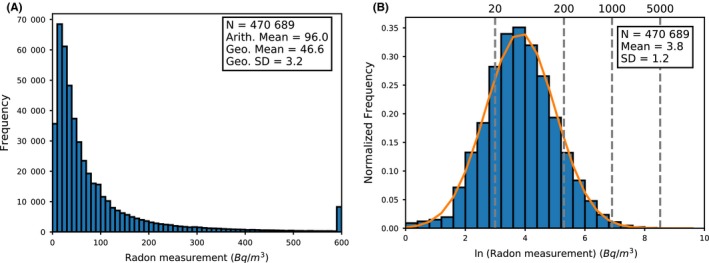
Distribution of radon measurements (A) for the full radon‐HEED dataset. The final bin is an overflow bin which contains all measurements above 600 Bq/m^3^. B shows the normalized histogram for the natural log of radon measurements fitted with a normal distribution (*μ* = 3.8, *σ* = 1.2). Dashed lines indicate thresholds at 20, 200, 1000, and 5000 Bq/m^3^ for guidance

Radon measured in the full sample (470 689 homes) is observed to be log‐normally distributed with a geometric mean of 46.6 Bq/m^3^ and an arithmetic mean of 96.0 Bq/m^3^ after subtracting for outdoor radon. Figure 3 shows the distribution of radon measurements for the full dataset.

### 3.1 Radon and dwelling characteristics

The geometric means and standard deviations are presented as a function of dwelling characteristics in Table 1. As mentioned in the methods section, substantial amounts of dwelling information in HEED are classed as “missing data”. For example, 42.7% of the homes are missing information on the type of dwelling. Despite this, the matched dataset is large enough to yield sufficiently sized samples. The data suggest that certain dwelling characteristics appear to be associated with higher average radon measurements. Bungalows are observed to have significantly higher radon levels than other dwelling types, while flats have the lowest. Older dwellings, in particular those built pre‐1900 tend to have higher radon concentrations and those built with sandstone or granite and whinstone walls have particularly high levels. These findings are consistent with those reported by Gunby et al^13^ There is limited linked data for newer dwellings (only 164 dwellings built post‐2003) due to the fact that newer homes are less likely to have undergone HEE retrofit.

**Table 1:** Geometric means and standard deviations for radon measurements grouped by dwelling type, age, wall type, number of bedrooms, and tenure

Dwelling variant	N homes (% total)	Arith. mean (Bq/m^3^)	Geo. mean (Bq/m^3^)	Geo. std. dev.
All homes	470 689	96.0	46.6	3.2
**Dwelling type**				
Missing data	201 032 (42.7)	96.5	46.2	3.2
End‐terrace	14 522 (3.1)	93.1	44.3	3.2
Mid‐terrace	27 747 (5.9)	82.8	40.7	3.2
Semi‐detached	65 772 (14.0)	77.4	39.6	3.1
Detached	101 919 (21.7)	100.3	49.5	3.2
Flat	12 995 (2.8)	79.5	36.8	3.3
Bungalow	46 691 (9.9)	123.9	63.0	3.1
**Age band**				
Missing data	232 162 (49.3)	96.2	46.1	3.2
Pre‐1900	21 222 (4.5)	128.3	61.9	3.2
1900‐1929	17 434 (3.7)	100.5	49.3	3.2
1930‐1949	23 590 (5.0)	87.7	45.5	3.1
1950‐1966	40 219 (8.5)	85.6	43.1	3.1
1967‐1975	51 435 (10.9)	94.3	45.6	3.2
1976‐1982	16 684 (3.5)	92.3	44.6	3.2
1983‐1990	17 947 (3.8)	92.7	48.3	3.0
1991‐1995	9847 (2.1)	86.2	43.5	3.1
1996‐2002	3112 (0.7)	78.2	43.8	2.9
2003‐2006	99 (0.0)	103.2	47.6	3.2
Post‐2006	65 (0.0)	76.3	47.7	2.8
Unknown	36 873 (7.8)	100.8	48.5	3.2
**Wall type**				
Missing data	279 291 (59.3)	96.3	46.3	3.2
Granite and whinstone	2060 (0.4)	134.8	57.7	3.7
Sandstone	12 142 (2.6)	149.2	75.9	3.1
Solid brick	17 145 (3.6)	92.4	46.3	3.2
Cavity	131 593 (28.0)	92.6	45.1	3.2
Modern timber frame	2912 (0.6)	87.9	43.6	3.4
Unknown walls	25 231 (5.4)	84.9	46.8	2.9
System built	315 (0.1)	76.8	24.9	4.2
**Number of bedrooms**				
Missing data	206 979 (44.0)	96.1	46.2	3.2
1	14 333 (3.0)	91.8	43.4	3.3
2	52 399 (11.1)	102.2	49.8	3.3
3	115 160 (24.5)	92.7	45.5	3.2
4	43 523 (9.2)	98.9	49.5	3.1
5+	16 688 (3.5)	102.3	50.2	3.2
Unknown	21 607 (4.6)	89.8	43.7	3.2
**Tenure**				
Missing data	216 824 (46.1)	95.7	46.1	3.2
Owner occupied	193 712 (41.2)	100.9	49.4	3.2
Privately rented	11 009 (2.3)	90.7	41.9	3.3
Rented from local authority	10 128 (2.2)	69.8	35.2	3.2
Rented from housing association	13 035 (2.8)	72.8	39.0	3.0
Social housing	2280 (0.5)	66.4	33.6	3.2
Other	729 (0.2)	125.6	59.0	3.1
Unknown	22 972 (4.9)	87.3	42.7	3.2

Note

“Missing data” refer to there being no entry in Home Energy Efficiency Database (HEED) or the radon measurement being pre‐HEED intervention, whereas “unknown” was flagged up this way by the building surveyor/data entry professional.

In terms of dwelling tenure, homes in the “other” category have the highest radon levels, although this is only a relatively small sample (~700 homes), which includes multi‐ownership properties such as care homes, second homes, and holiday rentals. Council and social housing properties are observed to have lower radon levels^23^—this may be because this tenure type is composed of a higher proportion of flats with no contact with the ground. Number of bedrooms appears to have little influence on radon levels. Radon concentrations by geographic location (region) and urban/rural class are shown in Appendix S2.

### 3.2 Radon and energy efficiency measures in homes

The full dataset was grouped by various HEE measures installed within homes (inclusive of all other interventions). The results presented in Table 2 indicate that homes that have undergone HEE interventions, tend to have higher average indoor radon measurements than those without. Homes with either a filled cavity or external insulation have higher radon levels than those with insulation as built. Note that the sample is small in the case for external insulation making it hard to draw firm conclusions regarding this intervention. Homes having any level of loft insulation all have higher radon levels than those with none. Double glazed homes are observed to have higher radon levels than those with only single glazing, while downdraft proofing appears to have little influence. Additional results showing radon measurements by heating system type are presented in Appendix S1.

**Table 2:** Geometric means and standard deviations for radon measurements grouped by home energy efficiency (HEE) measures for wall insulation, downdraft proofing, loft insulation, and glazing type inclusive of other HEE measures

EE dwelling variant	N homes (% total)	Arith. mean (Bq/m^3^)	Geo. mean (Bq/m^3^)	Geo. Std. dev.
All homes	470 689	96.0	46.6	3.2
**Wall insulation type**				
Missing data	385 771 (82.0)	90.9	44.7	3.2
External insulation	138 (0.0)	132.3	59.5	3.6
Filled cavity	22 495 (4.8)	151.9	67.8	3.5
Insulation as built	58 830 (12.5)	106.3	52.2	3.2
Insulation unknown	3412 (0.7)	123.1	59.2	3.2
Hybrid insulation	33 (0.0)	110.0	47.0	4.2
**Downdraft proofing**				
Missing data	422 209 (89.7)	92.3	45.0	3.2
None	36 583 (7.8)	120.6	60.5	3.2
Adequate	11 897 (2.5)	153.6	71.1	3.4
**Loft insulation level**				
Missing data	426 912 (90.7)	91.0	44.9	3.2
Unknown	6541 (1.4)	128.4	63.3	3.3
None	5198 (1.1)	113.1	52.3	3.4
1‐24 mm	377 (0.1)	165.3	77.3	3.4
25‐49 mm	1172 (0.2)	162.9	72.5	3.4
50‐74 mm	2476 (0.5)	140.6	67.9	3.4
75‐99 mm	2818 (0.6)	169.6	77.0	3.4
100‐149 mm	4168 (0.9)	153.0	71.7	3.4
150‐199 mm	3249 (0.7)	143.3	67.2	3.4
200‐249 mm	2049 (0.4)	153.2	66.4	3.6
250‐299 mm	15 245 (3.2)	154.2	72.8	3.4
300 mm+	484 (0.1)	122.9	54.7	3.4
**Glazing type**				
Missing data	408 526 (86.8)	91.0	44.7	3.2
Unknown	14 898 (3.2)	88.0	48.3	2.9
Single glazed	14 629 (3.1)	103.3	51.9	3.2
Double pre‐2002	22 234 (4.7)	147.7	68.7	3.4
Double post‐2002^a^	10 399 (2.2)	183.6	85.0	3.3

a Double glazing is split into pre‐ and post‐2002 to reflect the change in building regulations.^34^

### 3.3 Independent and combinations of energy efficiency measures

The results presented in Section 3.2 include homes inclusive of other energy efficiency interventions. In order to investigate the influence of individual and specific combinations of HEE measures, the data have been grouped for all combinations of intervention. To improve sample sizes, interventions are now classed as a binary variable (either present or not). Table 3 presents the geometric means and standard deviations for independent HEE interventions. The results here support findings presented in the previous section. Double glazing (Glz) is the intervention that has the single greatest influence on indoor radon, followed by the addition of loft (LI) and wall insulation (WI). Downdraft proofing (DP) appears to have less of an association with indoor radon and may in fact be associated with reduced levels in some cases, although this sample suffers from low statistics.

**Table 3:** Geometric means and standard deviations for radon measurements grouped by independent home energy efficiency interventions

Retrofit	N homes (% total)	Arith. mean (Bq/m^3^)	Geo. mean (Bq/m^3^)	Geo. std. dev.	% Change from “no recorded retrofit” case (95% CI)
All dwellings	470 689	96.0	46.6	3.2	NA
No recorded retrofit	419 754 (89.2)	89.4	44.4	3.2	NA
Downdraft proofing (DP)	346 (0.1)	98.7	49.7	3.2	12 (−8, 32)
Double glazing (Glz)	6899 (1.5)	159.3	74.1	3.4	67 (44, 89)
Loft Ins (LI)	8138 (1.7)	132.0	65.5	3.3	47 (26, 69)
Wall ins (WI)	6583 (1.4)	133.2	58.6	3.5	32 (11, 53)

**Figure 4:**
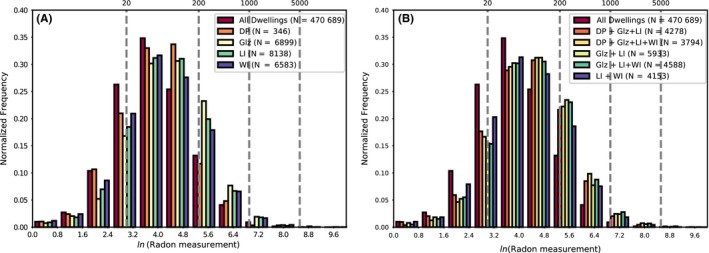
Normalized histograms showing ln(radon measurement) for independent (A) and combinations (B) of HEE measures. DP, downdraft proofing; Glz, glazing; LI, loft insulation; WI, wall insulation

Various combinations of HEE intervention are shown in Table 4, and histograms showing normalized distributions of ln(radon measurement) for a variety of HEE measures are shown in Figure 4. The results seem to suggest that interventions have a cumulative effect on radon levels, since a combination of all HEE interventions (DP + Glz + LI + WI), yield the highest geometric mean.

**Table 4:** Geometric means and standard deviations for radon measurements grouped by various combinations of home energy efficiency interventions

Retrofit combination	N homes (% total)	Arith. mean (Bq/m^3^)	Geo. mean (Bq/m^3^)	Geo. std. dev.	% Change from “no recorded retrofit” case (95% CI)
DP + Glz	1784 (0.4)	124.1	59.8	3.4	35 (13, 56)
DP + LI	850 (0.2)	133.6	61.8	3.6	39 (17, 61)
DP + WI	124 (0.0)	107.6	49.6	3.4	12 (−9, 32)
DP + Glz + LI	4278 (0.9)	159.8	72.3	3.5	63 (40, 85)
DP + Glz + WI	786 (0.2)	136.5	63.7	3.3	43 (22, 65)
DP + LI + WI	464 (0.1)	133.7	58.8	3.3	32 (12, 53)
DP + Glz + LI + WI	3794 (0.8)	170.7	80.7	3.3	82 (58, 105)
Glz + LI	5933 (1.3)	164.4	76.6	3.4	72 (50, 95)
Glz + WI	2215 (0.5)	159.9	67.0	3.5	51 (29, 73)
Glz + LI + WI	4588 (1.0)	175.8	79.6	3.4	79 (56, 103)
LI + WI	4153 (0.9)	139	63.2	3.5	42 (21, 64)

Abbreviations: DP, downdraft proofing; Glz, glazing; LI, loft insulation; WI, wall insulation.

**Figure 5:**
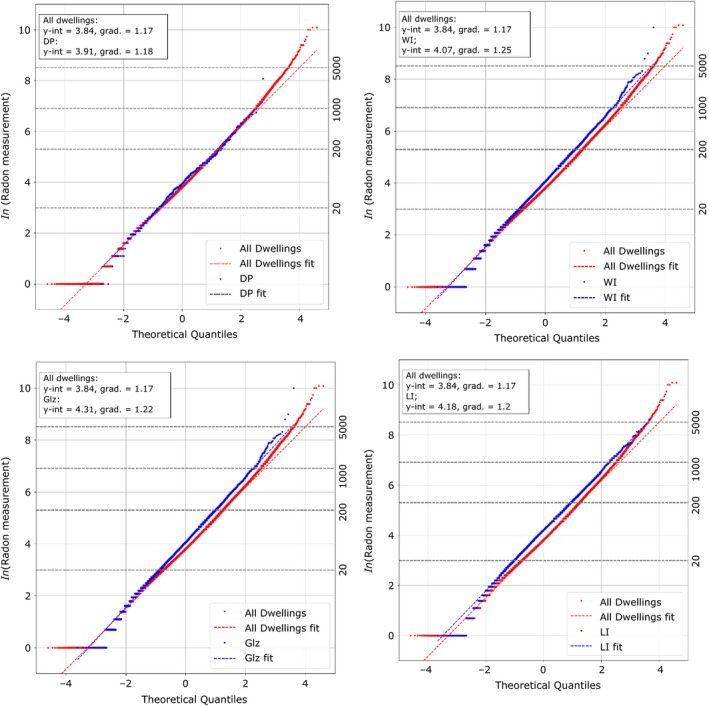
Quantile‐Quantile plots showing the ln(radon measurement) vs the theoretical quantiles for a normal distribution. Comparisons between the full dataset (all dwellings) and different independent home energy efficiency measures applied. Lines of the best fitting log‐normal distributions are shown for the data samples. DP, downdraft proofing; Glz, glazing; LI, loft insulation; WI, wall insulation

Quantile‐Quantile (Q‐Q) plots are shown in Figure 5 which test the log‐normality of the measured distributions. Comparisons are made between retrofit sub‐samples for glazing, loft and wall insulation and downdraft proofing and the full dataset. Dashed horizontal lines indicate radon concentrations of 20, 200, 1000, and 5000 Bq/m^3^, respectively. Deviations from log‐normality are observed at the lower end and tail of the distributions. These deviations are well understood and have been described elsewhere.^24^

## 4 DISCUSSION

This study shows that HEE measures that modify the building envelope and increase the airtightness of dwellings can have an adverse association with indoor radon levels. Double glazing was observed to have the single largest link with geometric mean of radon measurements, 67% (95% CI: 44, 89) higher than the “no recorded retrofit” case, closely followed by the addition of loft insulation (47%, 95% CI: 26, 69) and wall insulation (32%, 95% CI: 11, 53), while the association with downdraft proofing was less clear (12%, 95% CI: −8, 32). Multiple interventions appear to have a cumulative relationship with indoor radon, with homes having glazing combined with both loft and wall insulation having some of the highest levels, with a geometric mean 79% (95% CI: 56, 103) higher than homes with no recorded retrofit. Previous studies have shown that fabric retrofits can reduce air infiltration rates.^25^, ^26^, ^27^ Downdraft proofing, loft, and wall insulation have been observed to reduce air infiltration rates by anywhere between 24% and 71%.^27^ These studies all had small sample sizes, and to date, there has not been any large‐scale indoor air quality monitoring campaign and further research is required in this important subject area.

Our findings are consistent with those published by Gunby et al^13^ and indicate that dwellings with certain characteristics are more susceptible to higher radon levels. We find that bungalows were observed to have the highest average indoor radon measurements, while flats had the lowest. One might expect bungalows to have higher concentrations since both radon detectors will have been in ground floor rooms, where radon levels tend to be higher compared with the typical upstairs location of the bedroom in most houses. Flats meanwhile do not always have a direct connection with the ground floor in which case radon may only enter indirectly. Location may also have an influence with flats predominantly being in less radon‐prone urban areas. Older dwellings, particularly those built pre‐1900 with sandstone or granite and whinstone walls appear to be at a greater risk to high radon levels. Cracks in the foundations of older dwellings may have developed allowing radon to enter home more freely. Another possible reason may be that a higher proportion of older dwellings are built with basements and cellars. They also tend to have suspended wooden floors rather than concrete foundations which means that radon is able to enter the home more easily from the underlying soil.

Our results are in general agreement with findings in previous work which used a smaller dataset (N ~ 40 000) and showed that homes with double glazing have radon concentrations 66% higher than those without,^12^ similar studies exist in France and Switzerland.^28^, ^29^ Our findings add weight to previous *modeling* work which showed that the air tightening of the English housing stock could raise radon levels by an average of 57%.^11^ Milner et al went on to show that this increase could result in an annual burden of 4700 life years lost and 278 deaths (at peak). A greater level of public understanding surrounding the risks associated with radon exposure is required.^1^, ^30^, ^31^ Homeowners and HEE installers in radon‐affected areas^32^ should be aware of these risks and consider radon risk assessment/monitoring when performing a retrofit.

Fabric retrofits are intended to increase the thermal resistance of the building envelope; however, they also reduce ventilation rates allowing radon and other internally produced pollutants to accumulate. The resulting reduced airflow rates due to insulation may lead to maintaining a negative pressure gradient between indoors and outdoors which draws in more air through the floor. Downdraft proofing may have less of a modifying effect; since as well as preventing radon from leaving homes, it may in some cases act as a barrier to radon's entry. For example, if the retrofit involves installing hardboard across timber floors and applying sealing to skirting, this may have a beneficial impact on radon levels, while other types of downdraft proofing may be detrimental. HEED does not provide data on the types of downdraft proofing that were installed in homes which makes drawing any firm conclusions difficult.

In the UK, building regulations introduced in 2002 prescribe the levels of ventilation required to maintain a healthy indoor environment.^33^ Purpose provided ventilation (PPV) such as trickle vents and extract fans may be used to reach these minimum ventilation requirements. Mechanical Ventilation with Heat Recovery (MVHR) systems may also provide an effective means of preventing the buildup of harmful pollutants indoors.

### 4.1 Strengths, limitations, and future work

The main strength of this study is that it uses empirical data from a large sample (470 689) of UK homes measured over a long period of time (1980‐2015). It is the largest dataset of its kind and allows radon measurements from subsets of dwellings to be analyzed without the statistical limitations of smaller more controlled experiments. The analysis, however, was not without challenge and it is important to note, that despite that large sample size, a large amount of uncertainty remains due to this being a natural experiment over a long time frame.

The Homes Energy Efficiency Database contains only limited information on dwellings, a large proportion of which was missing (particularly for dwellings that had only undergone minor retrofit such as to lighting or heating controls). All homes with a radon measurement made prior to a HEED intervention are included in the “no recorded retrofit” sample used for base comparison. It is assumed that the majority of the homes in the “no recorded retrofit” sample have not had a HEE retrofit applied, which may be justified, since the average year in which radon measurements were made for these data sample is 1996 and HEE uptake did not become commonplace in the UK until the early 2000s.^18^ While HEED is estimated to have captured the majority of HEE activity during the period in question, unreported HEE retrofits including those made prior to HEED, changes in occupant behavior over time and other extraneous factors are likely to have introduced some hard to quantify uncertainties into our results. In the case of occupant behavior, for example, occupants of homes with HEE measures may behave differently to those without and behaviors are likely to have changed over the monitoring period. Within the dataset, there may also be biases regarding the types of homes that perform energy efficiency upgrades. Small sub‐sample sizes combined with confounding variables is the probable reason for some of the counterintuitive results observed in this study. Particularly in relation to combinations of measures with downdraft proofing where in some instances it appears DP is associated with lower geometric means, while in other cases they are not.

The National Energy Efficiency Database (NEED), which uses statistical techniques to determine missing data in HEED, may help address the issue of missing data. More detailed information such as on the presence of PPV and suspended floors would have helped answer further questions. In cases where there were multiple radon measurements for a dwelling, HEED information was matched to the first measurement. While this allowed the impact of HEE interventions to be investigated, it meant that homes having radon mitigation following a high radon reading were not considered. Future work will seek to examine how radon mitigation measures modify exposure on top of HEE interventions. There may also be opportunities to identify homes for follow‐up radon measurements where a measurement was made prior to, but not after retrofit.

As this was an observational study, it was not possible to control for various confounding variables. Unlike other studies,^12^ the required geographic location of homes to determine their underlying geology (radon potential) was not available. There is a known bias in the dataset toward higher radon areas (ie, most (51%) in the south west of England) due to the nature of the radon measurement campaigns conducted by PHE. An increase in geometric mean with year of radon measurement has been observed, with those measured post‐2000 within the “no recorded retrofit” base sample, having a geometric mean 33% higher than those pre‐2000. This may be partly attributed to this known bias in the radon measurement database, in particular the distribution over time of national and locally targeted surveys which may both introduce significant influences on the apparent trend over time. This bias has an important influence on the association of retrofit with radon, since retrofit sub‐samples have on average more recent radon measurements than the “no recorded retrofit” sample. This should be explored further, potentially by analyzing the distribution of radon measurements over time in bands of radon potential. This may identify whether the bias of the dataset toward properties in areas of elevated radon risk is a significant source of the apparent increase over time. The Wrixon et al^14^ study remains the only nationally representative analysis of radon exposure and is a study that needs repeating given how much has changed in the housing stock over that time. There are also limited data for homes built post‐2003 because few of these have retrofits. Future measurement campaigns could potentially target areas and dwelling types where data are currently sparse.

## 5 CONCLUSIONS

The matched Radon‐HEED dataset has provided a rich resource to observe, at a national level, how indoor radon concentrations vary with an increasingly energy‐efficient housing stock. The findings suggest that homes that have undergone certain fabric energy‐efficient retrofits are likely to have higher indoor radon concentrations than those without, which is likely to have consequences for other indoor pollutants. Double glazed windows were observed to have the largest association with indoor radon levels, 67% (95% CI: 44, 89) higher than dwellings with no recorded retrofit, while loft (47%, 95% CI: 26, 69) and wall insulation (32%, 95% CI: 11, 53) also have relatively strong associations. With an ever more energy‐efficient stock, this could result in a substantial rise in integrated population exposure and, hence, radon‐related lung cancer rates. This implies the importance of radon risk assessment and monitoring in conjunction with HEE improvements, especially in radon‐affected areas. The data matching process has helped identify homes that may be subject to further study. Obtaining additional radon measurements following a retrofit (where a prior radon measurement already exists) coupled with modeling work will further enhance our understanding of the relationship between HEE and indoor radon levels. This paper does not seek to discourage the installation of HEE measures. On the contrary, the UK must meet its carbon emission commitments to help mitigate anthropogenic warming of the climate and doing so while reducing exposure to indoor air pollutants will offer both climate change and health benefits.

## CONFLICT OF INTEREST

The authors have no conflict of interests to declare.
