# Supplementary material for: Home energy efficiency and radon: An observational study
Source: Indoor Air. 2019 Jun 18;29(5):854–64. doi: 10.1111/ina.12575 (PMC6772076; doi:10.1111/ina.12575)
Supplement: Supplementary file 1 [file INA-29-854-s001.docx]

doi: 10.1111/ina.12575

# **Supporting Information**

# A. Heating System Type and Indoor Radon

Table A1 presents geometric and arithmetic average indoor radon measurements across various heating system types recorded in HEED. Given the large variation in heating system type, it is difficult to establish if there is any clear link between heating system type and indoor radon levels. Further research is required to establish if any relationship exists between heating system and indoor radon.

| **Dwelling variant** | **N homes (% Total)** | **Arith. mean (Bq/m^3^)** | **Geo. mean (Bq/m^3^)** | **Geo. std. dev.** |
| --- | --- | --- | --- | --- |
| **Main Heating System** | | | | |
| Missing Data | 432933 (92.0) | 92.8 | 45.4 | 3.2 |
| Post-1998 Regular non-condensing | 969 (0.2) | 130.2 | 59.1 | 3.4 |
| Post-1998 Regular condensing | 4828 (1.0) | 141.7 | 66.8 | 3.3 |
| Post-1998 Non-condensing combi | 174 (0.0) | 164.0 | 71.7 | 3.6 |
| Post-1998 Condensing combi | 75 (0.0) | 280.0 | 77.5 | 4.4 |
| Pre-1998 fan flue Condensing | 864 (0.2) | 117.6 | 59.6 | 3.2 |
| Pre-1998 non-fan flue Wall mounted | 10885 (2.3) | 131.0 | 62.1 | 3.3 |
| 1979 to 1997 non-fan flue Floor mounted | 98 (0.0) | 154.6 | 73.1 | 3.3 |
| Pre-1998 non-fan flue Combi | 1088 (0.2) | 182.2 | 69.8 | 3.7 |
| Pre-1998 non-fan flue Back boiler | 404 (0.1) | 167.8 | 62.5 | 3.9 |
| Oil standard | 10112 (2.1) | 106.3 | 56.7 | 2.9 |
| Oil condensing | 183 (0.0) | 174.1 | 75.7 | 3.3 |
| Oil combi | 165 (0.0) | 183.4 | 88.8 | 3.2 |
| Solid Fuel | 1448 (0.3) | 95.0 | 47.7 | 3.2 |
| Electric | 440 (0.1) | 138.0 | 57.7 | 3.4 |
| Community heating | 26 (0.0) | 113.0 | 72.2 | 2.6 |
| Storage heaters | 4497 (1.0) | 172.7 | 80.9 | 3.4 |
| Warm Air | 318 (0.1) | 111.4 | 54.8 | 3.4 |
| GSHP | 10 (0.0) | 147.2 | 81.2 | 2.9 |
| Unknown | 89 (0.0) | 101.0 | 54.9 | 3.4 |

Table A1. Arithmetic and geometric mean radon measurements for various heating system types recorded in HEED. Homes where the radon measurement proceeded the HEED retrofit are classed as ‘Missing Data’. Abbreviation: GSHP – Ground Source Heat Pump.

# B. Regional and Urban/Rural Classification Variables

Table B1 presents the variability of radon measurements by region in the United Kingdom and by Urban/rural classification as defined by the Office for National Statistics (ONS) (ONS, 2011).

| **Dwelling variant** | **N homes (%)** | **Arithmetic mean (Bq/m^3^)** | **Geometric mean (Bq/m^3^)** | **Geometric std. dev.** |
| --- | --- | --- | --- | --- |
| **Region (GOR)** | | | | |
| Missing Data | 4 (0.0) | 32.5 | 30.4 | 1.5 |
| North East | 2010 (0.4) | 55.6 | 28.6 | 3.3 |
| South East | 18819 (4.0) | 76.7 | 40.8 | 3.0 |
| North West | 8768 (1.9) | 76.8 | 40.8 | 3.3 |
| Northern Ire. | 21089 (4.5) | 66.1 | 40.7 | 2.6 |
| West Mid. | 6507 (1.4) | 53.2 | 30.6 | 2.7 |
| Wales | 13545 (2.9) | 89.3 | 46.2 | 3.2 |
| South West | 240990 (51.2) | 119.7 | 60.2 | 3.1 |
| Scotland | 19039 (4.0) | 59.8 | 25.7 | 3.5 |
| London | 634 (0.1) | 30.5 | 17.0 | 3.0 |
| East of England | 3792 (0.8) | 29.8 | 20.6 | 2.4 |
| York and Humber | 6237 (1.3) | 52.6 | 29.3 | 3.0 |
| East Mid. | 129255 (27.5) | 74.1 | 36.0 | 3.2 |
| **Urban/Rural Classification** | | | | |
| Missing Data | 39535 (8.4) | 63.3 | 32.8 | 3.1 |
| Rural town and fringe (sparse setting) | 1858 (0.4) | 58.1 | 35.4 | 2.7 |
| Urban major conurbation | 2545 (0.5) | 24.8 | 14.1 | 2.9 |
| Urban city and town (sparse setting) | 1132 (0.2) | 49.6 | 32.8 | 2.5 |
| Rural village (sparse setting) | 4586 (1.0) | 81.3 | 46.0 | 2.8 |
| Rural hamlets and isolated dwellings | 125081 (26.6) | 119.2 | 58.1 | 3.2 |
| Rural hamlets and isolated dwellings (sparse setting) | 11355 (2.4) | 111.2 | 60.1 | 3.0 |
| Rural town and fringe | 29600 (6.3) | 108.6 | 54.3 | 3.3 |
| Rural village | 80289 (17.1) | 101.2 | 48.9 | 3.2 |
| Urban city and town | 172331 (36.6) | 84.1 | 41.8 | 3.1 |
| Urban minor conurbation | 2377 (0.5) | 33.9 | 19.5 | 2.8 |

Table B1. Geometric means and standard deviations for radon measurements grouped by region and urban/rural classification.

**References**

ONS, 2011 rural/urban classification. Office for National Statistics. 2011. [Online]. Available: https://www.ons.gov.uk/methodology/geography/geographicalproducts/ruralurbanclassifications/2011ruralurbanclassification. [Accessed 01 08 2018].
